# Le dermatofibrosarcome de Darier et Ferrand: à propos de 27 cas et revue de la literature

**DOI:** 10.11604/pamj.2014.18.280.1087

**Published:** 2014-08-06

**Authors:** Nawal Hammas, Ikram Badioui, Kaoutar Znati, Amal Benlemlih, Laila Chbani, Hind El Fatemi, Taoufiq Harmouch, Youssef Bouyahyaoui, Faouzi Boutayeb, Abdelmajid Mrini, Omar Mesbahi, Fatima Zahra Mernissi, Afaf Amarti

**Affiliations:** 1 Service d'anatomie pathologique, CHU Hassan II, Fès, Maroc; 2 Service de dermatologie, CHU Hassan II, Fès, Maroc; 3 Service de traumatologie-orthopédie, CHU Hassan II, Fès, Maroc; 4 Service d'oncologie, CHU Hassan II, Fès, Maroc

**Keywords:** Tumeur cutanée, dermatofibrosarcome de Darier et Ferrand, histopathologie, CD34, récidive, skin tumor, Darier-Ferrand dermatofibrosarcoma, histopathology, CD34, relapse

## Abstract

Le dermatofibrosarcome de Darier et Ferrand (DFS) est une tumeur mésenchymateuse cutanée de malignité intermédiaire. C'est une tumeur rare mais non exceptionnelle, représentant 0,1% des tumeurs cutanées malignes. Les auteurs présentent une étude rétrospective de 27 cas de DFS diagnostiqués sur une durée de 7 ans (2004 à 2010) et la comparent aux données de la littérature. Cette étude permet d’établir en plus des caractéristiques anatomopathologiques et immunohistochimiques, une étude épidémiologique, clinique et évolutive de ce sarcome. l’âge moyen de nos patients est de 41 ans avec une prédominance masculine. Le tronc est la localisation préférentielle touché dans 52% des cas. La taille tumorale a atteint 30cm et mesure en moyenne 6,1 cm. Le diagnostic était évoqué à l'examen histologique standard et confirmé par l'expression intense et diffuse du CD34. Le traitement était chirurgical, associé à une radiothérapie dans 2 cas. L’évolution était marquée par la transformation en un sarcome pléomorphe de haut grade dans un cas et par la survenue de récidives locales dans 2 cas. Nos résultats sont classiques et comparables aux autres séries de la littérature. L'examen histologique est indispensable pour le diagnostic. L'exérèse chirurgicale large est le traitement de référence. Le pronostic est conditionné par une malignité surtout locale et un fort potentiel de récidive. La transformation sarcomateuse franchement maligne métastasiante est exceptionnelle.

## Introduction

Le dermatofibrosarcome de Darier et Ferrand (DFS) est une tumeur mésenchymateuse cutanée maligne rare mais non exceptionnelle, représentant 0,1% des tumeurs cutanées malignes et moins de 5% des sarcomes des tissus mous de l'adulte [1–3]. Elle a été décrite par Jean Darier et Marcel Ferrand en 1924 [4]. Les sites de prédilection sont le tronc, suivi par les extrémités proximales puis la tête et le cou [3]. Le DFS touche souvent les patients dans leurs 3ème-4ème décades avec une légère prédominance masculine et se présente cliniquement sous forme d'une plaque ferme rougeâtre ou d'un nodule [5, 6]. Malgré sa présentation histologique distincte, son histogenèse reste indéfinie [3]. C'est une tumeur dite « à potentiel de malignité intermédiaire », de bon pronostic après résection complète, de croissance lente, à très haut risque de récidive locale, mais à potentiel métastatique faible [1, 2, 6, 7]. En raison de sa raret é, très peu d’études épidémiologiques lui ont été consacrées. Nous présentons une étude rétrospective de 27 cas de dermatofibrosarcome de Darier-Ferrand. Nous étudions les différentes caractéristiques de notre population et nous comparons nos données avec celles de la littérature.

## Méthodes

Nous avons réalisé une étude rétrospective portant sur 27 cas de dermatofibrosarcome de Darier Ferrand histologiquement prouvés, répertoriés au laboratoire d'anatomie pathologique du centre hospitalier universitaire Hassan II de Fès, durant une période de 7 ans (2004 à 2010). Nous avons analysé les caractéristiques épidémiologiques, cliniques, paracliniques, l'aspect anatomopathologique, la prise en charge thérapeutique et le suivi des patients. Le recueil de données est réalisé à partir des dossiers cliniques des patients et des renseignements cliniques accompagnant les prélèvements. Le diagnostic de dermatofibrosarcome de Darier Ferrand a été posé par examen histologique. Une confirmation immunohistochimique a été réalisée par les anticorps anti CD34, anti AML, anti-desmine, anti-H-caldesmone, anti PS100 et anti CD68.

## Résultats

Sur les 27 patients étudiés, nous avons retrouvé 17 hommes (63%) et 10 femmes (37%), soit un sexe ratio de 1,7 hommes pour une femme. La moyenne d’âge des patients était de 41 ans avec des extrêmes de 18 et 70 ans. La notion de traumatisme antérieur était retrouvée dans 2 cas, soit 7% des cas. Nous avons noté un seul cas associé à une grossesse. Le délai de diagnostic était long. Il variait entre 5 mois et 20 ans avec une moyenne de 8 ans. Il s'agissait d'une récidive dans 3 cas: une première récidive dans 2 cas et une deuxième récidive dans un cas.

**Figure 1:**
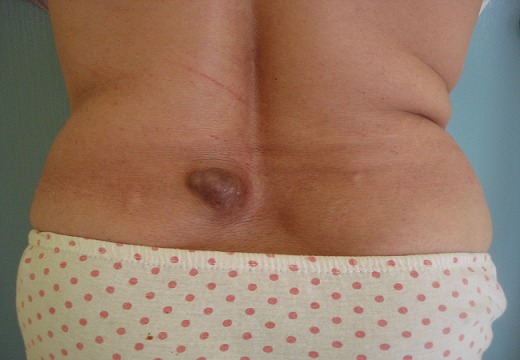
Lésion nodulaire du dos

Le tronc est la localisation préférentielle, touché dans 14 cas (52%), suivi par les membres inférieurs dans 6 cas (22%). La tête et le cou étaient touchés dans 4 cas (15%) et les membres supérieurs dans 3cas (11%). La taille tumorale variait de 0,8cm à 30cm avec une moyenne de 6,1cm. La tumeur était nodulaire dans 13 cas, multinodulaire dans 10 cas, et sous forme d'une plaque dans 4 cas (Figure 1). L'ulcération était notée dans 4 cas. La lésion était douloureuse dans 4 cas (15%). Les aires ganglionnaires étaient libres dans tous les cas et l’état général était altéré dans un seul cas.

**Figure 2:**
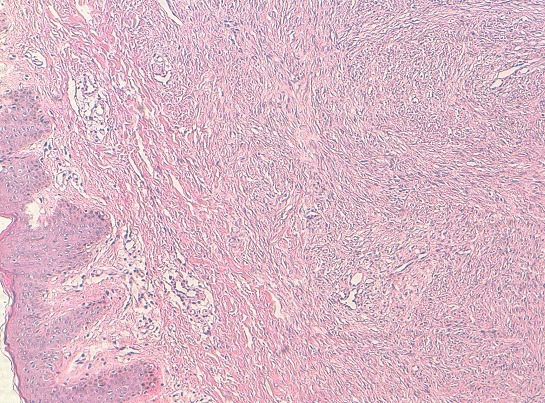
HES x 100: prolifération tumorale infiltrant le derme et l'hypoderme, d'architecture storiforme

**Figure 3:**
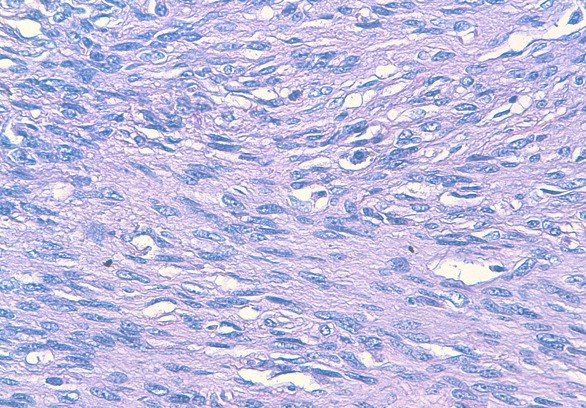
HES x 400: les cellules tumorales présentent des signes d'atypies nucléaires minimes à modérées

L'examen anatomopathologique a confirmé le diagnostic de DFS en montrant une prolifération dermo-hypodermique faite de faisceaux courts entrecroisés d'architecture storiforme, composés de cellules fusiformes uniformes peu ou modérément atypiques (Figure 2, Figure 3).

**Figure 4:**
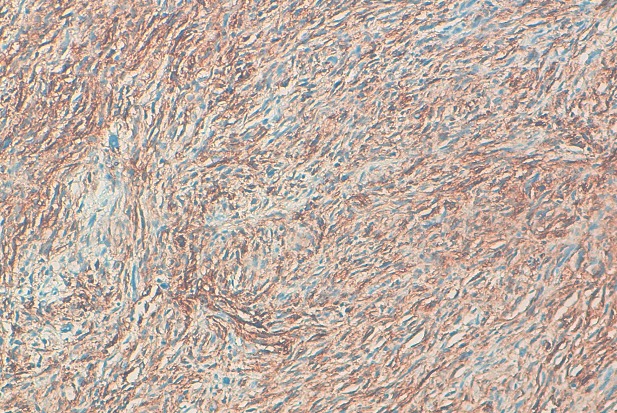
Positivité intense et diffuse par le CD34

La positivité de l'immunomarquage par le CD34 a confirmé le diagnostic dans tous les cas (Figure 4). L'immunomarquage par d'autres anticorps a permis d’éliminer les autres diagnostics différentiels notamment l'actine musculaire lisse réalisée dans 9 cas, l'H-caldesmone dans 3 cas, la desmine dans un cas, la PS100 dans 9 cas et le CD68 dans 3 cas. Tous ces marqueurs étaient négatifs.

Parmi les 27 cas étudiés, 25 cas (92,5%) étaient de grade 1 de la FNCLCC et seulement 2 cas étaient de grade 2. Parmi ces 2 cas, un cas a été transformé par la suite en un sarcome pléomorphe de grade 3.

Dans le cadre du bilan d'extension locorégional et a distance, une radiographie pulmonaire, une échographie abdominale, une TDM thoraco-abdomino-pelvienne et une IRM des parties molles ont été réalisées. Elles ont montré une infiltration tumorale des muscles sous jacents dans deux cas sans métastases à distance. Le traitement chirurgical a consisté en une exérèse large à 5 cm de la tumeur avec exérèse de l'aponévrose superficielle. La radiothérapie adjuvante était indiquée dans 2 cas. Une reprise a été nécessaire dans 6 cas suite à des limites de résection tumorales. Au cours du suivi des patients, nous avons noté 2 récidives locales sans aucun cas de métastases ganglionnaires ou à distance ou de décès.

## Discussion

Le DFS est une tumeur dermique mésenchymateuse de malignité intermédiaire [1]. C'est une tumeur rare mais non exceptionnelle, représentant entre 0,1% et 1% des tumeurs cutanées malignes [8]. Elle a été décrite pour la première fois en 1890 par Taylor [9] comme une tumeur sarcomateuse ressemblant à une cicatrice chéloïde, le dermatofibrosarcome fut rapporté ensuite par Kuznitzky et Grabish en 1921, Darier et Ferrand en 1924 [4], et Hoffman en 1925 [10] qui l'intitule « dermatofibrosarcoma protuberans ». En 1951, Pack et Tabah ont publié la première série importante [11] et en 1962, Taylor et Helwig ont établis les caractéristiques histologiques de cette entité [7, 12].

Notre étude permet d’établir en plus des caractéristiques anatomopathologiques et immunohistochimiques, une étude épidémiologique et clinique de ce sarcome. Nos données sont classiques et comparables aux autres séries de la littérature. La prédominance masculine que nous avons notée est conforme aux données de la majorité des auteurs. [2, 8, 12–14]

Comme décrit par plusieurs auteurs, cette tumeur peut survenir à n'importe quel âge avec des moyennes d’âge au moment du diagnostic oscillant entre 28 ans et 47 ans [8, 13, 14]. Le DFS est rare chez l'enfant de moins de 15 ans et la forme congénitale est exceptionnelle [15]. Dans notre série, l’âge moyen est de 41 ans et nous n'avons pas retrouvé de cas congénitaux ni de localisation chez l'enfant, le plus jeune patient avait 18 ans.

Le DFS peut toucher n'importe quelle partie du corps. Selon les données de la littérature, on note une prédilection pour le tronc qui est atteint dans 50 à 60% des cas. Les membres représentent 20 à 30% des localisations et 15 à 20% sont attribuées à la tête et au cou [11, 12, 14]. Dans notre série, la topographie correspond aux données de la littérature avec une atteinte préférentielle du tronc dans 52% et des membres dans 33% des cas. La localisation au niveau de la tête et du cou est de 15%. Nous n'avons pas noté de localisation au niveau des mains ni au niveau des pieds, comme publié par Smola [16].

Certains auteurs ont mentionné la survenue de DFS après un traumatisme local [17]. Cette notion est retrouvée dans 10 à 20% des cas [14, 18, 19]. Taylor et Helwig, dans une série incluant 115cas, retrouvent un antécédent de traumatisme dans 16,5% des cas [14]. Dans notre série, la survenue d'un traumatisme initial a été remarquée chez 2 patients soit 7% des cas.

Nous avons noté un cas diagnostiqué chez une femme enceinte. Il a été noté qu'au cours de la grossesse, le DDF présente une extension rapide ce qui suggère la présence de stimuli endocrines [7].

Le retard diagnostique est comparable à celui observé dans les autres études [2, 8, 11, 13, 14]. Le délai séparant l'apparition de la lésion et la première demande de soins dans notre série est de 8 ans. Ce retard est expliqué par l’évolution lente de la lésion et l'absence de signes fonctionnels et de troubles généraux [8].

Le diagnostic clinique est difficile. Au stade infiltratif, la lésion se présente comme une plaque indurée. A un stade plus avancé (stade nodulaire), la lésion d’étend réalisant au bout de quelques mois à quelques années, une masse multinodulaire. Cette évolution en deux stades n'est pas constante car certaines formes sont d'emblée uninodulaires ou multinodulaires. Non traitées, ces lésions peuvent devenir très volumineuses, ou bien s'ulcèrent pour devenir douloureuses et hémorragiques [2, 8, 11, 13, 14]. Dans notre série, la lésion était le plus souvent nodulaire ou multinodulaire.

Le caractère douloureux a été retrouvé chez 4 patients (15% des cas). Ce taux est superposable aux données de la littérature où la douleur est notée dans 10 à 25% des cas [12, 14]. Comme dans notre série, l’état général des patients est resté longtemps conservé [8]. L'altération de l’état général a été observée dans un seul cas dans notre série.

Selon les publications, la tumeur mesure en moyenne 1 à 5 cm [8, 14]. Des cas de “tumeurs monstrueuses ” atteignant 6,5 voire 7 kg ont été décrits [12]. Dans notre série, la taille moyenne est de 6,1cm. La taille maximale est de 30 cm, supérieure à celle de la série de Taylor (12cm) [14] et de Bédix-hansen (7cm) [11].

L'examen histologique est indispensable pour le diagnostic. La tumeur est faite d'une prolifération cellulaire dense, mal limitée, non encapsulée, occupant le derme, le plus souvent dans sa totalité. Elle envoie de fins prolongements parfois très profonds dans l'hypoderme, ce qui expliquerait la survenue de récidives même avec des marges de résection larges. L’épiderme est respecté. Les cellules sont allongées, fusiformes, à cytoplasme plus ou moins abondant, à noyau ovalaire, régulier. Les mitoses sont variables avec de rares atypies. Le stroma est variable d'une zone à l'autre.

Sur le plan architectural, les cellules sont disposées en faisceaux rayonnants (aspect en “rayon de roue”) ou tourbillonnants. Les zones nécrotiques sont rarement observées. [5, 8, 14, 20–22] Dans notre étude, nous avons noté les mêmes aspects histologiques décrits dans la littérature. La nécrose était notée dans un seul cas.

Peu d’études se sont intéressées au grade. Généralement, le DFS est considéré comme un sarcome de bas grade de malignité [8]. En effet, 92,5% des tumeurs de notre étude sont de grade 1 selon le grading de la FNCLCC. Bendix-Hansen et al, en utilisant le système de grading de Myhre-Jensen, ont constaté que, parmi les 19 cas étudiés, 15 sont de grade I, 4 sont de grade II et aucun cas n'est de grade III [11].

En général, l'aspect histologique permet de guider le diagnostic. Dans les cas douteux, l'immunohistochimie permet de distinguer le DFS des autres tumeurs à cellules fusiformes. Elle montre une positivité intense et diffuse du CD34, une positivité focale de l'AML (actine musculaire lisse) et une négativité constante de la desmine et de la PS100 [7]. Les zones en transformation sarcomateuse n'expriment qu'exceptionnellement et de façon très faible le CD34 [8]. Dans notre série, l'immunomarquage par le CD34 était intense et diffus. La négativité de l'immunomarquage par d'autres anticorps (AML, H-caldesmone, desmine, PS100 et CD68) a permis d’éliminer les autres diagnostics différentiels. Dans le cas où il y'avait une transformation en sarcome pléomorphe de haut grade, nous avons noté une négativation de l'immunomarquage par le CD34.

L'analyse cytogénétique montre la présence de chromosomes surnuméraires en anneau composés de séquences issues des chromosomes 17 et 22 ou plus rarement de translocations t(17;22) [20].

Le diagnostic différentiel se fait avec le neurofibrome diffus, la fasciite nodulaire, l'histiocytofibrome malin, le liposarcome myxoïde et le dermatofibrome. L'histiocytofibrome malin est caractérisé par un pléomorphisme marqué, une activité mitotique élevée et une nécrose intratumorale [7].

Le traitement est difficile en raison de l'extension infraclinique de la tumeur, pouvant être à l'origine d'une récidive. L'exérèse chirurgicale large est donc le traitement de référence avec des marges de sécurité de 4 à 5 cm et une ablation du fascia superficiel [5, 20–22]. La radiothérapie postopératoire est préconisée par certains, à partir de la seconde récidive [22]. La chimiothérapie systémique n'est pas recommandée [7]. Une surveillance clinique rigoureuse doit être maintenue, du fait de l’évolution lente et du haut pouvoir récidivant de cette tumeur [22]. Le traitement a été chirurgical dans notre série et a consisté en une exérèse large, avec ablation de l'aponévrose ou du fascia sous-jacent. La radiothérapie a été indiquée chez deux patients: le cas de transformation en sarcome pléomorphe de haut grade et le cas de la deuxième récidive.

Le pronostic est conditionné par une malignité surtout locale [5]. Le DFS ne donne quasiment jamais de métastases et l'envahissement ganglionnaire surviendrait dans moins de 1% des cas [7, 11, 13]. Son fort potentiel de récidive, malgré des exérèses chirurgicales souvent larges, transforme cette lésion en une entité difficilement contrôlable sur le plan clinique [7]. Pour de nombreux auteurs, la tendance à la récidive locale serait de 20 à 40% des cas [13]. La mort est exceptionnelle et survient tardivement par des complications locales [8]. Dans notre série, 3 cas étaient des récidives et au cours du suivi, nous avons noté deux cas de récidive. Par ailleurs, nous n'avons noté aucun cas de métastase ou de décès.

La transformation sarcomateuse franchement maligne métastasiante est exceptionnelle et se voit à un stade très tardif. [8] dans cette étude, un cas a été transformé en un sarcome pléomorphe de haut grade mais sans métastases.

## Conclusion

Intermédiaire entre l'inoffensif fibrome et le redoutable sarcome, le dermatofibrosarcome de Darier et Ferrand réalise une tumeur fibreuse rare de la peau qui se distingue par sa difficulté diagnostique, son évolution locale très lente, avec une tendance à la récidive locale avec de rares métastases. Il nécessite une surveillance clinique en raison de son haut pouvoir récidivant. Ses problèmes diagnostiques et thérapeutiques imposent un diagnostic histologique sûr confirmé par une étude immunohistochimique. La série étudiée présente des similitudes cliniques, histologiques et évolutives avec les données de la littérature.
